# Implementation and costs of a food insecurity resource navigation program for primary care patients with diabetes and hypertension in South Carolina

**DOI:** 10.1016/j.pmedr.2026.103434

**Published:** 2026-03-02

**Authors:** Deeksha Gupta, Darin Thomas, Stella Self, Edward A. Frongillo, Alain H. Litwin, Joseph A. Ewing, Lynnette Ramos-Gonzalez, A. Caroline Rudisill

**Affiliations:** a Department of Health Promotion, Education, and Behavior, Arnold School of Public Health, University of South Carolina, 915 Greene Street, Columbia, SC 29208, USA; b Addiction Medicine Center, Prisma Health, 701 Grove Road, Greenville, SC 29605, USA; c Department of Epidemiology and Biostatistics, Arnold School of Public Health, University of South Carolina, 915 Greene Street, Columbia, SC 29208, USA; d University of South Carolina School of Medicine, 607 Grove Road, Greenville, SC 29605, USA; e Data Support Core, Prisma Health, 605 Grove Road, Greenville, SC 29605, USA; f Accountable Communities, Prisma Health, 712 Grove Road, Greenville, SC 29605, USA

**Keywords:** Health-related social needs, Resource navigation, Chronic diseases, Implementation costs, Quality of life

## Abstract

**Objective:**

To examine food insecurity resource navigation program costs and how navigation intensity relates to clinical outcomes, healthcare costs, and quality of life (QOL) for diabetes and/or hypertension patients.

**Methods:**

This retrospective study included patients receiving resource navigation (July 12, 2021-December 31, 2022 with twelve-month follow-up) across three primary care practices in South Carolina's largest health system. Participants were 18+ years old (from electronic medical records/Epic), had food insecurity (from Hunger Vital Sign™), and diabetes and/or hypertension (from Epic registries). Matched controls came from food insecurity screening-only practices. Patients in each group (*n* = 219) had diabetes (9.13%), hypertension (50.23%), or both (40.64%). Navigation, fixed resources, and training costs were estimated using micro-costing from a US hospital system perspective (2025 USD). Multivariable models examined associations between outcomes (HbA1c, blood pressure, body mass index, primary, emergency, and inpatient charges, QOL), and navigation frequency, and duration, controlling for age, gender, race/ethnicity, payer, and comorbidities.

**Results:**

Program costs (total = $29,752.91) primarily included case reviews ($19,809.25), navigation ($5,068.67), and training ($1,975.84), averaging $135.86 per patient. Navigation intensity was associated with twelve-month changes in HbA1c, BMI, QOL, primary, and inpatient charges.

**Conclusions:**

Navigation programs incur significant personnel time costs. Individuals with great healthcare needs may require intensive navigation.

## Highlights

• Food insecurity resource navigation for diabetes/hypertension costs $135.86/patient.
• Personnel time for case reviews, navigation, and training drive program costs.
• Navigation intensity is linked to clinical outcomes and quality of life improvements.

## 1 Introduction

Health-related social needs (HRSNs) drive health inequities in individuals with chronic diseases. HRSNs, such as food insecurity, are associated with higher diabetes and hypertension risks than when individuals do not have these needs (Vazquez et al., 2021). Food access constraints can lead to poor diet quality due to reliance on cheaper, energy-dense foods (McCullough et al., 2022), increasing the risks of cardiometabolic conditions. Efforts to manage food insecurity often require trade-offs, including foregoing medication and medical care (Park et al., 2024), undermining disease management. Consequently, individuals with diabetes or hypertension experiencing food insecurity face increased risks of adverse health outcomes and higher healthcare expenses (Gucciardi et al., 2014; Berkowitz et al., 2018).

US health systems are increasingly screening patients for unmet HRSNs (Brewster et al., 2025) and collaborating with community-based organizations to address these needs using platforms and tools embedded in electronic medical records (EMRs) (Ruiz Escobar et al., 2021; Gupta et al., 2022). These screening and clinic-community collaboration models in healthcare settings, however, demonstrate mixed effects on health outcomes, healthcare use, and costs (Ruiz Escobar et al., 2021; Gottlieb et al., 2017). Among patients with diabetes and hypertension, stigma or embarrassment around food assistance, low perceived need, limited food literacy or cooking skills, high cost of nutritious foods, long enrollment times, and insufficient dietary resources (DePuccio et al., 2022; Stotz et al., 2021; Creevy and Boocock, 2026), may reduce referral uptake. Provider-related factors such as time constraints, lack of resources knowledge, and referral follow-up uncertainty (DePuccio et al., 2022; Creevy and Boocock, 2026) can also restrict food-related resource connections, necessitating coordinated care between healthcare and community resources.

The American College of Preventive Medicine recommends integrating navigation with HRSN screening and referral processes to overcome implementation and resource access barriers (Livingston et al., 2025). Resource navigation (hereafter ‘navigation’) involves connecting patients with unmet HRSNs to community-based services through education, referral coordination, and follow-up. Navigators can be community health workers (CHWs) (Enard and Ganelin, 2013; Esperat et al., 2012; Mirambeau et al., 2013) or trained laypersons (Loskutova et al., 2016) collaborating with clinical teams to ensure that HRSNs are addressed as a part of comprehensive care. Prominent navigation programs such as the Accountable Health Communities Model and North Carolina's Healthy Opportunities Pilots program have reduced healthcare use and spending for Medicaid and Medicare beneficiaries (Parish et al., 2023; Berkowitz et al., 2025). Regular navigator interaction also boosts patient trust in health systems by addressing needs beyond medical care (Bryant et al., 2020). Existing studies describe navigation intensity (e.g. contact duration, frequency) (Loskutova et al., 2016; Mercer et al., 2019; Krieger et al., 2005; Peek et al., 2024), but few examined its relationship with patient outcomes (Mercer et al., 2019). Navigation cost studies are also limited, primarily reporting salaries and fringe benefits (Enard and Ganelin, 2013; Krieger et al., 2005; Johnson et al., 2012).

We address this research gap by (1) examining how navigation intensity (contact duration and frequency) relates to clinical outcomes (HbA1c, blood pressure, body mass index (BMI)), healthcare charges (primary care, emergency department, inpatient), and quality of life (QOL), and (2) identifying, measuring, and valuing the costs of providing food insecurity navigation to diabetes and/or hypertension patients.

## 2 Methods

### 2.1 Study design and population

#### 2.1.1 Navigation program

We implemented HRSNs screening and navigation in three primary care practices with high diabetes and hypertension caseloads within Prisma Health, South Carolina's largest non-profit health system. Located in the Upstate (northwest) region of South Carolina, the practices included two urban and one rural, and two internal medicine and one family medicine specialty sites. In 2021, the Upstate's 813,069 residents were 75.8% White, 14.6% Black/African-American, 6.5% Hispanic, 14.2% in poverty (vs. 11.4% nationally) and 13.9% uninsured (vs. 10.2% nationally) (Prisma Health, 2022).

Patients were screened in-person for food insecurity with the Hunger Vital Sign™ questions. “Sometimes true” or “often true” responses indicated a positive screen (Hager et al., 2010). HRSN screener responses are reported elsewhere (Gupta et al., 2025). Adults (18+ years) having food insecurity and diabetes and/or hypertension (identified from Epic registries) were eligible for navigation from July 12, 2021 to December 31, 2022 with twelve-month follow-up.

The navigation program and its main outcomes have been described previously (Gupta et al., 2025). The program employed a single navigator trained in Epic (the health system's EMR platform) and screening processes. The navigator developed the navigation processes, participant engagement and case management with the project lead (DT), including documentation and implementation. Participants were informed about local resources (e.g., food pantries, food banks, utilities support) via text, email, or mail. The navigator discussed resource access barriers and mitigation strategies, following up every 2–3 weeks for twelve months to address new barriers or needs. Patients could also initiate contact. Case summaries (e.g., social needs, access barriers, strategies discussed) were reviewed before each call to tailor outreach. Patients were considered lost to follow-up after up to five unsuccessful contact attempts, including to the next-of-kin.

#### 2.1.2 Study sample

Of 7592 patients screened for food insecurity, 371 (4.89%) were program-eligible and 236 (3.11%) consented. Controls were drawn from practices conducting HRSN screening without navigation, matched using 1:1 propensity scores on patient's age, gender, race/ethnicity, BMI, payer, food insecurity status, screening date, and practice specialty (family/internal medicine). Matching was done within diabetes, hypertension, and combined diabetes and hypertension subgroups.

### 2.2 Measures

#### 2.2.1 Clinical outcomes, healthcare charges, QOL, and independent variables

Outcomes included: (1) HbA1c for diabetes, (2) blood pressure for hypertension, (3) BMI, (4) primary care, (5) emergency department, and (6) inpatient charges, and (7) QOL. The EMR-based clinical outcomes were recorded during visits and assessed at baseline (i.e., screening) and twelve-month follow-up, within an eight-week window before and after each timepoint. Baseline data came from records closest to screening within the eight-week window or, if unavailable, the nearest date before screening. Follow-up data were the closest data to twelve months within the window.

The EMR-based healthcare charges included billed amounts for services during visits (e.g., treatment, physician time, room/board, supplies, overheads). Zero charges were assigned if no visits occurred. Baseline and twelve-month follow-up charges were charges accrued in the 26 weeks (∼six months) before each time point, capturing all visits near the study time points.

The navigator collected QOL data at baseline, six and twelve months follow-ups using the Euroqol 5-Dimension 5-Level (EQ-5D-5L) questionnaire, measuring five health dimensions: mobility, self-care, usual activities, pain/discomfort, and anxiety/depression (Herdman et al., 2011). Responses ranged from ‘no problems’ to ‘unable to/extreme problems’ and were summarized into an EQ-5D-5L score using US population valuation weights (Pickard et al., 2019). EQ-5D-5L scores vary between <0 (0 representing death; <0 a health state worse than death) and 1 (‘full health’).

Independent variables included navigation contact frequency and duration from call logs. Models adjusted for EMR-based patient age, gender, race/ethnicity, primary payer, and number of comorbidities. Comorbidities including cancer (heart, colorectal, prostate, lung, endometrial), chronic pulmonary disease, heart failure, mental health disorder, asthma, Alzheimer's, depression, substance use (alcohol and drug use) were defined using Chronic Conditions Data Warehouse ICD-10 codes (Center for Medicare and Medicaid Services, 2023). Cerebrovascular disease was defined using Charlson Comorbidity Index ICD-10 codes (Gasparini, 2018). Patients were identified as having these conditions if they had any defining criteria over the study period.

#### 2.2.2 Navigation program measures

Navigation variables included: (1) a binary indicator for high frequency of successful calls, and (2) mean duration per call per patient. High call frequency was defined as per patient successful call count (i.e., when navigator connected with patients) exceeding the sample mean, calculated as total successful calls divided by number of patients. Mean duration per call per patient was calculated over twelve months and categorized as <6 minutes, 6–8 minutes, and 8+ minutes. Controls, who did not receive navigation, were assigned a call frequency and duration of 0.

### 2.3 Statistical analyses

#### 2.3.1 Estimating the relationship between navigation intensity and patient outcomes

Regression models with individual-specific random effects ($\delta_{i}$) and random errors (${∊}_{\mathrm{it}}$) examined the relationship between navigation intensity and outcomes ($Y_{\mathrm{it}}$: clinical outcomes, healthcare charges and QOL):

$$Y_{\mathrm{it}}=\beta_{0}+\beta_{1}\mathrm{HFRE}Q_{i}+\beta_{2}\mathrm{DURATIO}N_{i}+\beta_{3}\mathrm{TIM}E_{\mathrm{it}}+\beta_{4}\left(\mathrm{DURATIO}N_{i}\times \mathrm{TIM}E_{\mathrm{it}}\right)+\beta_{5}\mathrm{GROU}P_{i}+\beta_{6}\mathrm{DE}M_{i}+\delta_{i}+{∊}_{\mathrm{it}}$$

Navigation variables included the high call frequency indicator ($\mathrm{HFRE}Q_{i}$,) and mean duration per call per patient ($\mathrm{DURATIO}N_{i}$). $\mathrm{TIM}E_{\mathrm{it}}$ captured the time effects (baseline vs. twelve-month follow-up) on outcomes. The interaction term, $\mathrm{DURATIO}N_{i}\times \mathrm{TIM}E_{\mathrm{it}}$, depicted outcomes changes over twelve months by call duration. The group indicator, $\mathrm{GROU}P_{i}$, was excluded from QOL models, as QOL data were not collected for controls. Covariates included a vector of patient demographics ($\mathrm{DE}M_{i}$): age, gender, race/ethnicity, payer, and number of comorbidities.

Two-part models assessed healthcare charges, accounting for excess zero-charge observations. The first part modeled the likelihood of incurring any charge (vs. none) using a logit specification. The second part modeled positive charges using generalized estimating equations, with error distributions determined from the modified Park test (Deb et al., 2017). A gamma distribution was used for primary care charges, and an inverse Gaussian distribution for inpatient and emergency department charges, each with a log link.

After model fitting, average marginal effects of $\mathrm{DURATIO}N_{i}\times \mathrm{TIM}E_{\mathrm{it}}$ were calculated to assess variation in outcomes by call duration over twelve months. Predicted means (for continuous outcomes) and probabilities (for binary healthcare charges) were also estimated. Results describe associations between patient outcomes and navigation intensity, without implying causality.

#### 2.3.2 Estimating navigation program costs

We estimated navigation program costs using micro-costing, following the Consolidated Health Economic Evaluation Reporting Standards checklist (Husereau et al., 2022). A US hospital system perspective was adopted to incorporate costs borne by health systems in providing navigation services.

We calculated average navigation program cost as the total program cost divided by number of participants. Program costs were estimated for (1) navigation-related activities (e.g., case reviews, follow-up calls, travel to primary care sites), (2) fixed resources (e.g., mobile phones, cell phone plan, HRSN screening and referral platform access), and (3) navigator training. Total costs across resource categories were estimated by multiplying volume for each resource by its unit cost (Jones et al., 2019). Training and activity costs were valued at the average wage rate and fringe for a navigator and units estimated from interviews with the navigator and program lead and call logs. Non-labor unit costs and volume also came from interviews. Costs are reported in 2025 USD.

This study was approved by the Institutional Review Board at Prisma Health (IRB no. Pro00105924). Analyses were performed using Stata/MP v.19 and Microsoft Excel.

## 3 Results

### 3.1 Study sample and characteristics

**Fig. 1:**
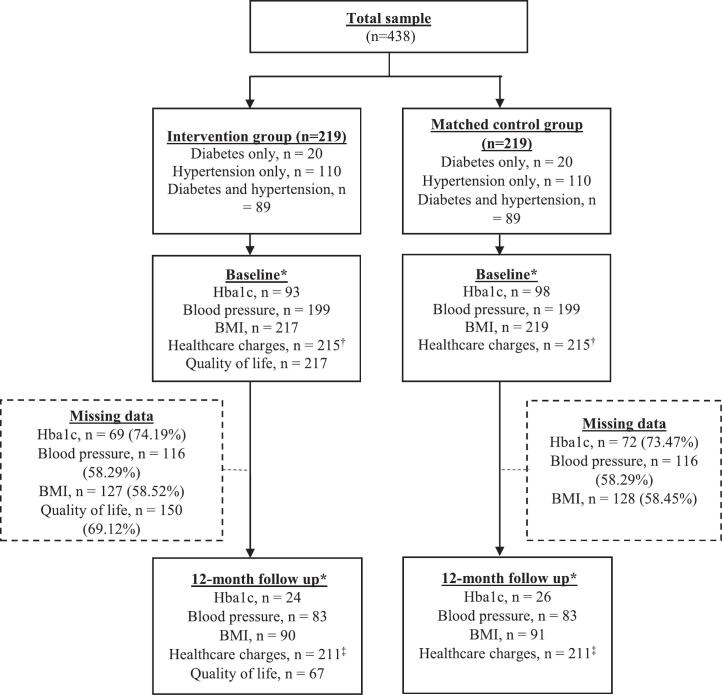
*Baseline and twelve-month for EMR-based data (HbA1c, blood pressure, BMI) were defined using a window of 8 weeks before and after the study timepoint. 'Baseline' data was determined as the EMR data captured nearest to the screening date within this window. If no baseline EMR data met this criterion, the closest available data before the screening date was considered 'baseline'. Similarly, the EMR data closest to the twelve-month follow-up after screening within the 8-week window was designated as 'follow-up' data. †Patients whose healthcare charges at baseline fell within the top 1% in each group were identified as outliers and subsequently excluded from the analysis, along with their corresponding matched counterpart (n = 8). ‡Patients whose healthcare charges at twelve-month follow up fell within the top 1% in each group were excluded from the analysis along with their matched counterparts (n = 8).

Of 219 patients in each of the navigator and control groups, 20 (9.13%) patients had diabetes, 110 (50.23%) hypertension, and 89 (40.64%) had both (Fig. 1). Clinical data were missing at both baseline and twelve-month follow-up. QOL data was collected from only the navigator group with data missing at both baseline and follow-up. Baseline demographics, clinical outcomes, and QOL were compared between patients with complete vs. missing follow-up outcomes data within the full sample, navigator, and control groups (Appendix Tables A.1-A.4). No differences were observed for the following groups: all patients and navigator group. Controls with missing follow-up HbA1c data had lower HbA1c (7.05 ± 1.43% vs. 7.82 ± 2.19%; *p* = 0.05) and age (51.82 ± 15.10 vs. 59.81 ± 10.31 years; *p* = 0.01) than patients with complete data. Fewer controls with missing BMI or blood pressure data had 3+ comorbidities than those with complete data.

Baseline demographics and outcomes for controls are discussed elsewhere (Gupta et al., 2025). Navigator group patients were aged 55.00 ± 13.75 years, with 144 (65.75%) female patients, 98 (44.75%) Black/African American, 60 (27.52%) having private/commercial payer, 68 (31.19%) having Medicaid, and 58 (26.61%) Medicare (Table 1). Patients had one (46; 21.00%), two (78; 35.62%), or 3+ comorbidities (95; 43.38%).

**Table 1:** Baseline navigation program features, clinical outcomes, healthcare charges, and demographics of participants (n = 219) in a resource navigator program in South Carolina, July 12, 2021 to December 31, 2022 with 12-month follow-up.

Variables			n (calls or patients)	Mean (SD)/Percent	Range
*Patient demographics*					
Age (in years)			219	55.00 (13.75)	20–91
				
Gender			219		
	Male		75	34.25%	
	Female		144	65.75%	
				
Race/ethnicity			219		
	Black or African American		98	44.75%	
	Other race†		121	55.25%	
				
Primary payer			218		
	Private/commercial‡		60	27.52%	
	Medicaid§		68	31.19%	
	Medicare||		58	26.61%	
	Other payer¶		32	14.68%	
				
Number of comorbidities			219		
	1		46	21.00%	
	2		78	35.62%	
	3+		95	43.38%	
				
*Clinical outcomes at baseline*					
HbA1c (%)			93	7.73 (2.25)	4.90–15
Systolic BP (in mmHg)			199	134.61 (19.24)	90–184
Diastolic BP (in mmHg)			199	81.63 (14.32)	23–136
BMI			217	35.08 (9.68)	15.12–84.08
			
*Healthcare charges at baseline (in 2023 USD)*					
Primary care charges			215	$387.71 (319.04)	$0–$2,212.00
Emergency department charges			215	$284.21 (623.85)	$0–$5,596.00
Inpatient charges			215	$6,167.67 (18,896.22)	$0–$109,319.20
			
*Quality of life*					
EQ-5D-5L score at baseline			217	0.66 (0.31)	−0.29 - 1
			
*Resource navigation program features*					
Call initiator⁎			3842		
	Contact attempt by navigator		3627	94.40%	
	Contact attempt by patient		215	5.60%	
				
Number of calls, mean per patient⁎			3842	17.58 (11.75)	3–66
	Number of unsuccessful calls⁎		2008	52.26%	
		No answer/busy	1885	93.87%	
		Missing or invalid number	93	4.63%	
		Left message	20	1.00%	
		Not available	10	0.50%	
	Calls with missing data on call outcomes⁎		1834	47.74%	
				
Number of successful calls, mean per patient			1644	7.51 (5.56)	1–27
Contact duration per successful call (in minutes)			1644	7.49 (6.14)	0–57
Total contact duration per patient (in minutes)			1644	56.22 (43.11)	10–253
				
Contact duration per successful call per patient (in minutes)			219	8.45 (4.33)	3.33–50
	0–6 minutes		56	25.57%	
	6–8 minutes		77	35.16%	
	More than 8 minutes		86	39.27%	

Note: BP = =blood pressure, BMI = body mass index, EQ-5D-5L = Euroqol 5-Dimension 5-Level

⁎ *n* = 218 due to missing data for one patient.

† Other race includes White (*n* = 104), Hispanic (*n* = 11), American Indian or Alaska Native, Native Hawaiian or Other Pacific Islander, Asian, and individuals with two or more races (*n* = 4), patient refused/unknown race (n = 2).

‡ Private/commercial includes BlueCross BlueShield and Commercial.

§ Medicaid includes Medicaid and Medicaid MCO (*n* = 43), and Managed Care (*n* = 25).

|| Medicare includes Medicare (*n* = 26), and Medicare Advantage (*n* = 32).

¶ Other payer includes other, self-pay, pending Medicaid, and Tricare.

### 3.2 Baseline clinical outcomes, healthcare charges, and QOL for navigator group

At baseline, the navigator group had a mean HbA1c of 7.73 ± 2.25% for patients with only diabetes or both diabetes and hypertension, systolic blood pressure (SBP) of 134.61 ± 19.24 mmHg, diastolic blood pressure (DBP) of 81.63 ± 14.32 mmHg for patients with only hypertension or both diabetes and hypertension, and BMI of 35.08 ± 9.68 (Table 1) for all patients. Average healthcare charges in the year prior to baseline were: primary care ($387.71 ± 319.04), emergency department ($284.21 ± 623.85), and inpatient charges ($6,167.67 ± 18,896.22). Baseline EQ-5D-5L score was 0.66 ± 0.31.

### 3.3 Navigation program features

Of 3842 calls, 3627 (94.40%) were navigator-initiated and 2008 (52.26%) were unsuccessful (Table 1). Unsuccessful calls resulted from non-response or busy lines (93.87%), missing or invalid numbers (4.63%), voicemails (1.00%) or patient unavailability (0.50%). Call outcome data were missing for the remaining 1834 calls.

As successful calls were not recorded, calls with missing outcome data but any duration were deemed as ‘successful’. Of 1834 calls with missing outcome, 1644 were ‘successful’. Successful calls per patient was 7.51 ± 5.56, each lasting an average 7.49 ± 6.14 minutes. Over twelve months, the navigator spent an average of 56.22 ± 43.11 minutes total per patient on calls. The mean duration per call per patient was 8.45 ± 4.33 minutes, reflecting the average call length for each patient.

### 3.4 Relationship between patient outcomes and navigation intensity

Call frequency was not associated with clinical outcomes and QOL (Table 2). At baseline, clinical outcomes did not differ by navigation contact duration (Table 3). Decrease in HbA1c over twelve months was associated with <6-minute calls (−0.44[SE = 0.22]; *p* = 0.04), but not longer calls. At twelve months, HbA1c did not differ by contact duration. BMI decreases over twelve months were associated with 6–8 minute calls (−1.00[0.50]; *p* = 0.05). At twelve months, BMI was higher for 8+ minute vs. <6-minute calls (4.06[1.75]; *p* = 0.02). Twelve-month changes in SBP and DBP had *p* > 0.05. Both outcomes were lower for 6–8 minute vs. <6-minute calls at twelve months: SBP (−10.51[4.19]; *p* = 0.01) and DBP (−6.57[2.56]; p = 0.01).

**Table 2:** Associations between 12-month changes in clinical outcomes, quality of life and resource navigator intensity for resource navigation program participants and matched controls in South Carolina, July 12, 2021 to December 31, 2022 with 12-month follow-up.

		**(1)**	**(2)**	**(3)**	**(4)**	**(5)**
Variables		HbA1c Mean (Std. err.)	SBP Mean (Std. err.)	DBP Mean (Std. err.)	BMI Mean (Std. err.)	QOL^i^ Mean (Std. err.)
Contact frequency (ref. = low)						
	High	0.32	3.03	1.30	1.15	−0.07
		(0.49)	(2.56)	(1.78)	(1.32)	(0.04)
					
Mean contact duration (ref. <6 minutes)						
	6–8 minutes	−0.85	−4.50	−4.13	0.87	−0.15**
		(0.67)	(3.43)	(2.38)	(1.57)	(0.05)
	8+ minutes	−1.09	−0.25	1.52	3.35	−0.17**
		(0.67)	(3.33)	(2.25)	(1.73)	(0.05)
						
Time (ref. = baseline)						
	12 months	−0.44*	0.33	−0.99	−0.30	0.09*
		(0.22)	(1.66)	(0.93)	(0.28)	(0.04)
						
Mean contact duration × time (ref. <6 minutes × baseline)						
	6–8 minutes × 12 months	0.47 (0.32)	−6.01 (3.83)	−2.44 (2.33)	−0.70 (0.58)	−0.00 (0.07)
	8+ minutes × 12 months	1.01** (0.36)	−3.25 (3.75)	−1.43 (2.36)	0.71 (0.51)	−0.08 (0.08)
						
Group (ref. = control)						
	Intervention	1.17* (0.60)	3.66 (3.11)	1.24 (2.07)	−2.34 (1.59)	–
						
*Patient demographics*						
Age		−0.02	−0.04	−0.24**	−0.29**	−0.00
		(0.01)	(0.07)	(0.05)	(0.04)	(0.00)
						
Gender (ref. = female)						
Male		0.03 (0.32)	4.13* (1.81)	1.91 (1.20)	−3.25** (0.98)	−0.00 (0.04)
						
Race/ethnicity (ref. = Black/African American)						
	Other race†	−0.19	−1.69	−2.02	−1.73	−0.09*
		(0.32)	(1.72)	(1.11)	(0.93)	(0.04)
						
Primary payer (ref. = Private/commercial‡)						
	Medicaid§	−0.08	4.60*	3.21*	−2.04	−0.05
		(0.38)	(2.07)	(1.49)	(1.18)	(0.04)
	Medicare||	−0.35	1.59	−2.66	1.46	−0.04
		(0.38)	(2.53)	(1.62)	(1.28)	(0.05)
	Other payer¶	−0.83	2.31	1.55	−1.22	−0.06
		(0.46)	(2.54)	(1.70)	(1.62)	(0.06)
						
No. of comorbidities (ref. = 1)						
	2	0.01	−0.43	−1.42	2.88*	−0.15**
		(0.54)	(2.42)	(1.57)	(1.21)	(0.05)
	3+	0.23	−3.29	−3.29*	2.38*	−0.14**
		(0.53)	(2.35)	(1.47)	(1.19)	(0.041)
						
Constant		7.48**	130.59**	82.86**	35.89**	1.01**
		(0.54)	(2.38)	(1.51)	(1.23)	(0.06)
						
No. of observations		247	564	564	617	282
No. of patients		197	398	398	436	216
Wald $\chi^{2}$		$\chi^{2}$(15) = 24.92	$\chi^{2}$(15) = 31.04	$\chi^{2}$(14) = 138.61	$\chi^{2}$(15) = 92.29	$\chi^{2}$(14) = 61.78
Prob > $\chi^{2}$		0.05	0.01	0.00	0.00	0.00

Note: SBP = systolic blood pressure, DBP = diastolic blood pressure, BMI = body mass index, QOL = quality of life.

Standard errors in parentheses.

** p < 0.01, * p < 0.05.

† Other race includes White, Hispanic, American Indian or Alaska Native, Native Hawaiian or Other Pacific Islander, Asian, and individuals with two or more races, patient refused/unknown race.

‡ Private/commercial includes BlueCross BlueShield and Commercial.

§ Medicaid includes Medicaid and Medicaid MCO, and Managed Care.

|| Medicare includes Medicare, and Medicare Advantage.

¶ Other payer includes other, self-pay, pending Medicaid, and Tricare.

i Model includes only intervention group patients as QOL data was collected from exclusively this group. A group indicator variable was therefore not applicable in this model specification.

**Table 3:** Marginal effects of mean contact duration on predicted clinical outcomes, quality of life, and healthcare charges (from two-part models) over 12 months for resource navigation program participants and matched controls in South Carolina, July 12, 2021 to December 31, 2022 with 12-month follow-up.

Outcomes		Average-adjusted predicted means (SE)		Between group difference Mean (SE)		Within-group difference Mean (SE)
		Baseline	12 months	Baseline	12 months	
HbA1c (%)						
	<6 mins	7.89 (0.28)	7.45 (0.31)	ref.	ref.	−0.44 (0.22)*
	6–8 mins	7.04 (0.48)	7.07 (0.52)	−0.85 (0.67)	−0.379 (0.705)	0.03 (0.23)
	8+ mins	6.80 (0.48)	7.37 (0.52)	−1.09 (0.67)	−0.081 (0.714)	0.57 (0.29)
						
SBP (mmHg)						
	<6 mins	133.81 (1.45)	134.14 (1.76)	ref.	ref.	0.33 (1.66)
	6–8 mins	129.32 (2.67)	123.63 (3.48)	−4.50 (3.43)	−10.51 (4.19)*	−5.69 (3.43)
	8+ mins	133.57 (2.51)	130.64 (3.46)	−0.25 (3.33)	−3.50 (4.16)	−2.93 (3.37)
						
DBP (mmHg)						
	<6 mins	81.61 (0.92)	80.62 (1.05)	ref.	ref.	−0.99 (0.93)
	6–8 mins	77.48 (1.91)	74.05 (2.08)	−4.13 (2.38)	−6.57 (2.56)*	−3.43 (2.14)
	8+ mins	83.13 (1.72)	80.71 (2.42)	1.52 (2.25)	0.09 (2.84)	−2.42 (2.17)
						
BMI						
	<6 mins	34.74 (0.70)	34.44 (0.71)	ref.	ref.	−0.30 (0.28)
	6–8 mins	35.61 (1.20)	34.60 (1.14)	0.87 (1.57)	0.17 (1.53)	−1.00 (0.50)*
	8+ mins	38.08 (1.36)	38.49 (1.38)	3.35 (1.73)	4.06 (1.75)*	0.41 (0.42)
						
QOL						
	<6 mins	0.77 (0.04)	0.86 (0.04)	ref.	ref.	0.09 (0.04)*
	6–8 mins	0.62 (0.04)	0.71 (0.05)	−0.15 (0.05)**	−0.16 (0.05)**	0.09 (0.06)
	8+ mins	0.61 (0.04)	0.62 (0.07)	−0.17 (0.05)**	−0.24 (0.07)**	0.02 (0.08)
						
Primary care charges – first part, odds ratio						
	<6 mins	0.92 (0.02)	0.39 (0.04)	ref.	ref.	−0.53 (0.03)**
	6–8 mins	0.90 (0.04)	0.39 (0.07)	−0.02 (0.04)	−0.00 (0.09)	−0.51 (0.06)**
	8+ mins	0.86 (0.04)	0.28 (0.06)	−0.06 (0.05)	−0.12 (0.08)	−0.59 (0.06)**
						
Primary care charges – second part (in 2023 USD)						
	<6 mins	$594.43 (22.18)	$545.87 (30.25)	ref.	ref.	−$48.57 (36.24)
	6–8 mins	$557.52 (46.54)	$654.67 (73.97)	−$36.91 (52.85)	$108.81 (78.53)	$97.16 (77.10)
	8+ mins	$617.77 (59.60)	$583.57 (77.59)	$23.33 (65.29)	$37.70 (83.01)	−$34.20 (87.85)
						
Emergency department charges – first part, odds ratio						
	<6 mins	0.28 (0.03)	0.10 (0.02)	ref.	ref.	−0.18 (0.03)**
	6–8 mins	0.32 (0.06)	0.13 (0.04)	0.04 (0.08)	0.03 (0.05)	−0.18 (0.07)**
	8+ mins	0.43 (0.07)	0.15 (0.04)	0.15 (0.08)	0.04 (0.05)	−0.28 (0.07)**
						
Emergency department charges – second part (in 2023 USD)						
	<6 mins	$1,046.26 (177.60)	$840.95 (165.61)	ref.	ref.	−$205.31 (170.43)
	6–8 mins	$693.12 (156.42)	$784.11 (201.96)	−$353.14 (281.01)	−$56.84 (294.82)	$90.98 (200.34)
	8+ mins	$569.06 (77.31)	$1,349.81(483.05)	−$477.20 (225.40)*	$508.86 (534.69)	$780.75 (462.68)
						
Inpatient charges – first part, odds ratio						
	<6 mins	0.09 (0.02)	0.06 (0.02)	ref.	ref.	−0.03 (0.02)
	6–8 mins	0.15 (0.04)	0.06 (0.08)	0.06 (0.05)	0.00 (0.03)	−0.09 (0.05)
	8+ mins	0.07 (0.03)	0.05 (0.03)	−0.02 (0.04)	−0.01 (0.03)	−0.02 (0.04)
			
Inpatient charges – second part (in 2023 USD)						
	<6 mins	$62,343.35 (10,535.16)	$31,326.37 (3099.97)	ref.	ref.	−$31,016.98 (9401.55)**
	6–8 mins	$38,130.31 (5832.46)	$50,270.03 (11,485.66)	−$24,213.03 (13,458.96)	$18,943.66 (11,891.91)	$12,139.72 (11,904.76)
	8+ mins	$42,830.41 (8967.94)	$54,735.52 (12,219.60)	−$19,512.94 (16,253.41)	$23,409.15 (13,111.00)	$11,905.11 (14,141.94)

Note: BMI=Body mass index, DBP = diastolic blood pressure, SBP = systolic blood pressure, QOL = quality of life.

** p < 0.01, * p < 0.05.

At baseline, compared to <6-minute calls, QOL was lower for 6–8 minute (−0.15[0.05]; p = 0.01) and 8+ minute calls (−0.17[0.05]; *p* < 0.01) (Table 3). At twelve months, QOL remained lower for 6–8 minute (−0.16[0.05]; *p* < 0.01) and 8+ minute calls (−0.24[0.07]; *p* < 0.01) than <6-minute calls. QOL increase over twelve months was associated with <6-minute calls (0.09[0.04]; p = 0.02) but not longer calls.

Contact frequency was not associated with probability of incurring healthcare charges, except primary care charges (Table 4). Patients having above-average call volumes were 5.54 (SE = 2.64; p < 0.01) times more likely to incur primary care charge than those having low call volumes. The decrease in odds of primary care charges (Table 3) from baseline to twelve months was associated with all contact durations: <6 minutes (odds ratio =0.92[SE = 0.02] vs. 0.39[0.04]; *p* < 0.01), 6–8 minutes (0.90[0.04] vs. 0.39[0.07]; p < 0.01), and 8+ minutes (0.86[0.04] vs. 0.28[0.06]; p < 0.01). Decrease in emergency department charges probability from baseline to twelve months was also associated with all contact durations: <6 minutes (0.28[0.03] vs. 0.10[0.02]; *p* < 0.001), 6–8 minutes (0.32[0.06] vs. 0.13[0.04]; p < 0.01) and 8+ minutes (0.43[0.07] vs. 0.15[0.04]; p < 0.01). Inpatient charges probability did not change during this period.

**Table 4:** Two-part models depicting associations between healthcare charges and navigation intensity among resource navigation program participants and matched controls in South Carolina, July 12, 2021 to December 31, 2022 with 12-month follow-up.

Variables		(1)		(2)		(3)	
		Primary care charges		Emergency department charges		Inpatient charges	
		First part, Odds ratio#	Second part, coeff.††	First part, Odds ratio#	Second part, coeff.‡‡	First part, Odds ratio#	Second part, coeff.‡‡
Contact frequency (ref. = low)							
	High	5.54 (2.64)**	0.04 (0.08)	1.17 (0.41)	0.12 (0.19)	1.75 (0.74)	0.04 (0.20)
Contact duration (ref. < 6 min)							
	6–8 minutes	0.71 (0.49)	−0.06 (0.09)	1.25 (0.60)	−0.41 (0.33)	1.99 (1.26)	−0.49 (0.26)
	8+ minutes	0.43 (0.28)	0.04 (0.11)	2.41 (1.17)	−0.61 (0.26)*	0.75 (0.51)	−0.38 (0.32)
Time (ref. = baseline)							
	12 months	0.02 (0.01)**	−0.09 (0.07)	0.21 (0.06)**	−0.22 (0.18)	0.55 (0.20)	−0.69 (0.15)**
Mean contact duration × time (ref. <6 minutes × baseline)							
	6–8 minutes × 12 months	1.41 (0.86)	0.25 (0.14)	1.16 (0.65)	0.34 (0.32)	0.51 (0.38)	0.97 (0.30)**
	8+ minutes × 12 months	1.09 (0.64)	0.03 (0.16)	0.67 (0.35)	1.08 (0.38)**	1.17 (0.98)	0.93 (0.32)**
Group (ref. = control)							
	Intervention	0.38 (0.19)	−0.42 (0.08)**	1.18 (0.51)	0.29 (0.25)	1.16 (0.68)	0.14 (0.28)
*Patient demographics*							
Age		1.00 (0.01)	0.01 (0.00)**	0.96 (0.01)**	−0.00 (0.01)	0.98 (0.01)	0.02 (0.01)**
Gender (ref. = female)							
	Male	0.84 (0.23)	−0.06 (0.05)	1.21 (0.32)	−0.28 (0.12)*	1.45 (0.49)	−0.14 (0.15)
Race/ethnicity (ref. = Black/African American)							
	Other†	0.79 (0.21)	0.03 (0.05)	0.95 (0.22)	0.13 (0.12)	1.76 (0.59)	0.15 (0.20)
Primary payer (ref. = Private/commercial‡)							
	Medicaid§	1.03 (0.33)	0.03 (0.07)	1.14 (0.36)	−0.09 (0.17)	1.97 (0.93)	−0.37 (0.21)
	Medicare||	0.62 (0.24)	−0.10 (0.08)	2.87 (1.04)**	0.13 (0.22)	2.72 (1.52)	−0.31 (0.27)
	Other payer¶	1.55 (0.63)	0.01 (0.09)	1.73 (0.64)	−0.07 (0.19)	4.56 (2.37)**	−0.25 (0.28)
No. of comorbidities (ref. = 1)							
	2	0.90 (0.32)	0.12 (0.07)	0.76 (0.25)	0.14 (0.17)	1.52 (0.77)	−0.49 (0.24)*
	3+	1.38 (0.47)	0.30 (0.06)**	1.47 (0.45)	0.23 (0.15)	3.86 (2.00)**	−0.48 (0.23)*
Constant		43.20 (26.34)**	6.39 (0.08)**	0.15 (0.06)**	6.61 (0.15)**	0.01 (0.00)**	11.59 (0.30)**
No. of observations		842	538	842	182	842	65
No. of patients Wald $\chi^{2}$		421	385	421	154	421	57
		$\chi^{2}$(15) = 67.37	$\chi^{2}$(15) = 142.94	$\chi^{2}$(15) = 62.79	$\chi^{2}$(15) = 31.51	$\chi^{2}$(15) =31.22	$\chi^{2}$(15) =111.17
Prob > $\chi^{2}$		0.00	0.00	0.00	0.01	0.01	0.00

Standard errors in parentheses.

** *p* < 0.01, * *p* < 0.05.

† Other race includes White, Hispanic, American Indian or Alaska Native, Native Hawaiian or Other Pacific Islander, Asian, and individuals with two or more races, patient refused/unknown race.

‡ Private/commercial includes BlueCross BlueShield and Commercial.

§ Medicaid includes Medicaid and Medicaid MCO, and Managed Care.

|| Medicare includes Medicare, and Medicare Advantage.

¶ Other payer includes other, self-pay, pending Medicaid, and Tricare.

# Model includes all healthcare charges and is estimated using a logit model with clustered standard errors.

†† Model includes healthcare charges>0 and is estimated using generalized estimated equation with a gamma distribution, log link function, and robust standard errors.

‡‡ Model includes healthcare charges>0 and is estimated using generalized estimated equation with an inverse Gaussian distribution, log link function, and robust standard errors.

Among patients incurring any healthcare charges (second part), baseline emergency department charges differed by contact duration, but primary care and inpatient charges did not (Table 3). Patients with 8+ minute calls had lower emergency department charges than <6-minute calls (−$477.20[225.40]; *p*=0.03). Over twelve months, positive primary care and emergency department charges remained unchanged, while decrease in inpatient charges was associated with <6-minute calls (−$31,016.98[9401.55]; p<0.01). At twelve months, primary care, emergency department, and inpatient charges did not differ by contact duration.

### 3.5 Sensitivity analyses

Robustness of complete-case regressions were assessed with multiple imputation. Missing baseline clinical outcomes and QOL data were imputed using chained (sequential) equations with 10 imputed datasets, and patient characteristics as predictors. Imputations for missing follow-up data included baseline outcomes as additional predictors. Results were consistent with the complete-case analysis, showing no association between navigator contact frequency and patient outcomes (Appendix Table A.5). However, associations between contact duration and twelve-month changes in some outcomes (HbA1c, QOL) were smaller in magnitude with higher standard errors in the multiple imputation models (Appendix Table A.6).

### 3.6 Navigation program costs

Cost estimates covered the entire 903-day program duration, including 538 days of patient recruitment (July 12, 2021 to December 31, 2022) and 365-days follow-up. Total implementation cost was $29,752.91 (range = $19,815.65–$70,451.04), averaging $135.86 ($90.48–$321.69) per patient (Table 5). Costs for navigation activities included case reviews ($19,809.85; range= $15,055.49–$24,960.42), successful calls ($5,068.67; $0.000–$40,502.00) and travel to practices ($308.73; $293.29–$324.16). Fixed costs included a mobile phone ($500.00), cell phone plan ($1,335.94), and HRSN technology platform access ($753.88). Navigator training cost $1,975.84 ($1,877.05–$2,074.63).

**Table 5:** Implementation components and total program costs for a resource navigation program in South Carolina, July 12, 2021 – December 31, 2022 with 12-month follow-up ⁎(2025 USD).

Program component	No. of units (Q)	Cost per unit (P)	Total cost (P x Q)	Source
***Navigation-related activities***				
Case review time cost (Time: 0.208 (0.167–0.250) hours per case)	3850 case reviews‡	$5.15§ ($3.91 - $6.48)	$19,809.85 ($15,055.49 - $24,960.42)	Team members (navigator, program lead) interviews, program operational data (call logs)
				
Resource navigation time cost (Time: 0.125 (0–0.95) hours per call)	1644 calls	$3.08§ ($0.00 - $24.64)	$5,068.67 ($0.00 - $40,502.00)	Team members interviews (navigator, program lead), program operational data (call logs)
				
Resource navigator travel time cost (Time: 0.833 hours per trip) – 903 days	15 trips	$20.58§ ($19.55 - $21.61)	$308.73 ($293.29 - $324.16)	Team members (navigator, program lead) interviews
				
***Fixed resources***				
Mobile phone - 903 days	1	$500.00	$500.00	Team members (program lead) interviews
Cell phone plan - 903 days	1	$1,335.94	$1,335.94	Team members (program lead) interviews
Social needs technology platform access† - 538 days	1	$753.88	$753.88	Health system
***Resource navigator training cost***	80 hours	$24.70 ($23.46 - $25.93)	$1,975.84 ($1,877.05 – $2,074.63)	Team members (navigator, program lead) interviews
**Total program costs (903 days)**			**$29,752.91** **($19,815.65 - $70,451.04)**	
				
**Program cost per patient (n = 219)**			**$135.86** **($90.48 - $321.69)**	

⁎ Cost estimates are based on 903 days, which includes social needs screening dates (538 days; July 12, 2021 – December 31, 2022) with 12-month follow-up until December 31, 2023 (365 days).

† Includes one-off costs for health system to start technology platform usage such as integration with electronic medical record and incorporation of health-related social needs screening survey tool. Assumes maximum number of health system users allowed at pricing tier (*n* = 499).

‡ Calculation of number of case reviews for each patient: If number of all call attempts is greater than or equal to number of successful calls, number of case reviews = number of all contact attempts. If number of all contact attempts is less than number of successful calls, number of case reviews = number of successful calls.

§ Cost calculations based on average resource navigator wage (including fringe): $24.70/hour (range = $23.46 – $25.93).

## 4 Discussion

A primary care-based food insecurity navigation program for diabetes and/or hypertension patients demonstrated a cost per patient that may be reasonable for further implementation in healthcare settings. Also, navigation intensity was associated with twelve-month changes in HbA1c, BMI, QOL, and healthcare charges, but not blood pressure.

Navigation programs vary in delivery modality, personnel involved, and intensity. A layperson-led phone-based navigation providing social, food, and transportation support to primary care-based at-risk or diabetes patients averaged 6.1 calls per patient over nine months (Loskutova et al., 2016). In a socially deprived region in Scotland, primary care-based community links practitioners providing unlimited in-person navigation demonstrated QOL improvements and reduced anxiety and depression over nine months after 3+ meetings (Mercer et al., 2019). Referral uptake declined, however, after four meetings. A CHW-led housing navigation for families of children with asthma involving seven hourly visits for a year improved caregiver QOL and reduced urgent care use compared to families receiving single visit with limited resource information (Krieger et al., 2005). An East Oakland-based navigation program for patients with diabetes and HRSNs involved 26.7 contact attempts over 9.1 months, primarily for housing, food insecurity, poverty and other material needs (Peek et al., 2024). Compared to these programs, our navigation program had higher contact frequency (17.6 contact attempts per patient; 7.51 successful calls per patient) with shorter duration (7.49 minutes per call, 0–57 minutes range) (Krieger et al., 2005).

Our findings show associations between navigation intensity and patient outcomes, but causality cannot be claimed due to observational design. At baseline, patients with <6-minute calls had higher QOL than longer calls, with no differences for HbA1c or BMI. Shorter calls (<6-minutes and 6–8 minutes) were linked to HbA1c and BMI decreases and QOL improvements over 12 months, but longer calls showed no associations. These patterns suggest a bidirectional relationship between navigation intensity and patient outcomes. Navigation may contribute to improvements in outcomes, while patients' health status may, in turn, influence navigation intensity, with longer calls reflecting greater patient needs (e.g., lower QOL). Factors such as health literacy, social support, and disease severity may also influence both navigation intensity and outcomes, confounding these associations.

The likelihood of primary care and emergency department charges decreased over twelve months, indicating that navigation may have improved patients' ability to manage health, reducing acute care use and costs. Decrease in inpatient charges were associated with <6-minute calls, but not longer calls. By addressing resource access barriers with consistent, tailored support, navigators can build patient trust and confidence in navigating health systems and seeking medical care (Dahrouge et al., 2022). It is possible that patients incurring inpatient charges had severe underlying conditions and could access needed services with intensive navigation. Overall, these findings further highlight the bidirectionality of navigation intensity and patient outcomes, warranting future studies and program designs that consider how patient features affect resources needed from the navigation services.

Few studies report navigation costs or cost-related outcomes. A CHW-led navigation for 1905 emergency department-discharged patients addressing HRSNs (e.g., transportation, financial resources, childcare) cost $45,880 (2013 USD) annually, reflecting CHW salary and fringe benefits (Enard and Ganelin, 2013). Another Texas-based CHW-led navigation for patients with multimorbidity cost $6,360.8 (2012 USD) per patient (Esperat et al., 2012). A navigation program for high-utilizers reported $559 annual per-patient costs from CHW salary and service delivery (Johnson et al., 2012). In contrast, a three CHW-led navigation in rural Vermont included broader costs for start-up, operations, training, and personnel, costing $420,640 (2013 USD) annually and $140,116 per CHW (Mirambeau et al., 2013). Our navigator program had substantially lower total ($29,752.91) and per-patient costs ($135.86).

Our study has limitations. First, we could not assess the incremental cost-effectiveness ratio (ICER) of the navigation program. QOL data were not collected for controls, precluding incremental effectiveness comparisons. Although, the absence of ICER limits drawing definitive conclusions about program cost-effectiveness, our cost findings can inform future economic evaluations of navigation programs. Second, findings are restricted to primary care practices in a specific region within one health system, restricting generalizability to other healthcare settings (e.g., emergency or inpatient) where navigation intensity may differ. Third, due to observational design and the lack of QOL data for controls, causal navigation effects on outcomes cannot be ascertained. Moreover, navigation intensity was assumed constant, but may have varied over time (i.e., higher initially and tapering as patients gain stability), affecting outcomes. Experimental studies may elicit longitudinal effects of varying navigation intensity on outcomes. Fourth, we could not include six-month outcomes data in our assessment (Gupta et al., 2025). The navigation data included call episodes for the entire twelve-month intervention period but not associated dates, precluding assessment of the program's temporal effects. Finally, follow-up clinical outcomes and QOL data had substantial missingness as data were collected during visits. We found no differences in demographics and clinical outcomes between patients with complete vs. missing data, except among controls. Controls with missing data were younger, had better glycemic control and fewer comorbidities than those with complete data. These patterns suggest that healthier controls may have had fewer visits, contributing to missingness. Importantly, since associations between outcomes and navigation intensity were assessed for the full sample, missingness is unlikely to bias comparative findings. Sensitivity analyses using multiple imputation were also consistent with complete-case models, with smaller effects and higher standard errors for some outcomes (Appendix Tables A.5-A.6).

## 5 Conclusion

US health systems are increasingly implementing screening protocols to identify patients' HRSNs (Brewster et al., 2025) and connecting them to community-based resources. Referral uptake remains inconsistent across systems, however, with higher engagement observed when navigation services are available (Ruiz Escobar et al., 2021). We demonstrate that a primary care-based navigation incurs lower costs than existing programs, even when considering broader cost categories, warranting investigating program cost-effectiveness. Although cost-effectiveness cannot be ascertained, our findings can inform future economic evaluations of resource navigation programs. Experimental studies are also needed to understand longitudinal effects of navigation features on patient outcomes.

## Data sharing statement

Data are available from Prisma Health, but restrictions apply to the availability of these data, which were used under agreement for the current study, and so are not publicly available. De-identified data are, however, available from the authors upon reasonable request and with permission of Prisma Health.

## CRediT authorship contribution statement

**Deeksha Gupta:** Writing – review & editing, Writing – original draft, Visualization, Methodology, Formal analysis, Data curation. **Darin Thomas:** Writing – review & editing, Supervision, Resources, Project administration, Investigation, Funding acquisition, Conceptualization. **Stella Self:** Writing – review & editing, Supervision, Methodology, Funding acquisition, Formal analysis, Data curation. **Edward A. Frongillo:** Writing – review & editing, Methodology. **Alain H. Litwin:** Writing – review & editing, Methodology. **Joseph A. Ewing:** Writing – review & editing, Resources, Data curation. **Lynnette Ramos-Gonzalez:** Writing – review & editing, Investigation. **A. Caroline Rudisill:** Writing – review & editing, Supervision, Resources, Project administration, Methodology, Funding acquisition, Data curation, Conceptualization.

## Funding declaration

This research was funded by The Duke Endowment. The funder has no role in design and conduct of the study; collection, management, analysis, and interpretation of data; preparation, review or approval of the manuscript or the decision to submit the manuscript for publication.

## Declaration of competing interest

The authors declare the following financial interests/personal relationships which may be considered as potential competing interests: Deeksha Gupta, A. Caroline Rudisill, Darin Thomas, Stella Self, and Lynnette Ramos-Gonzalez report financial support was provided by The Duke Endowment. Deeksha Gupta reports a relationship with Prisma Health that includes: funding grants and travel reimbursement. Deeksha Gupta reports a relationship with The Duke Endowment that includes: travel reimbursement. Darin Thomas reports a relationship with The Duke Endowment that includes: travel reimbursement. Darin Thomas reports a relationship with Community Health Worker Association-Credential Council that includes: board membership. Darin Thomas reports a relationship with Challenges Inc. SC that includes: board membership. Darin Thomas reports a relationship with SC Bar Association Lawyers helping Lawyers that includes: board membership. Darin Thomas reports a relationship with Mental Health America Greenville County that includes: board membership. Stella Self reports a relationship with Prisma Health that includes: funding grants. Stella Self reports a relationship with National Institutes of Health that includes: funding grants. Stella Self reports a relationship with The Companion Animal Parasite Council that includes: funding grants and travel reimbursement. Stella Self reports a relationship with Centers for Disease Control and Prevention that includes: funding grants. Stella Self reports a relationship with South Carolina Department of Health and Environmental Control that includes: funding grants. Stella Self reports a relationship with US Health Resources and Services Administration that includes: funding grants. Stella Self reports a relationship with Merck that includes: speaking and lecture fees. Alain H Litwin reports a relationship with Gilead Sciences that includes: consulting or advisory, funding grants, and travel reimbursement. Alain H Litwin reports a relationship with AbbVie that includes: consulting or advisory. Alain H. Litwin reports a relationship with Tenvos that includes: equity or stocks. Lynnette Ramos-Gonzalez reports a relationship with The Duke Endowment that includes: funding grants and travel reimbursement. A. Caroline Rudisill reports a relationship with National Institute of Diabetes and Digestive and Kidney Diseases that includes: funding grants. A. Caroline Rudisill reports a relationship with Viiv Healthcare that includes: funding grants. A. Caroline Rudisill reports a relationship with South Carolina Center for Rural and Primary Healthcare that includes: funding grants. A. Caroline Rudisill reports a relationship with Prisma Health that includes: funding grants. A. Caroline Rudisill reports a relationship with Centers for Disease Control and Prevention that includes: funding grants. If there are other authors, they declare that they have no known competing financial interests or personal relationships that could have appeared to influence the work reported in this paper.

## Data Availability

Data are available from Prisma Health, but restrictions apply to the availability of these data, which were used under agreement for this study, and so are not publicly available. De-identified data are, however, available from the authors upon reasonable request and with permission of Prisma Health.

## Appendix A Supplementary data

Supplementary material Image 1
