# Supplementary material for: Implementation and costs of a food insecurity resource navigation program for primary care patients with diabetes and hypertension in South Carolina
Source: Prev Med Rep. 2026 Mar 2;64:103434. doi: 10.1016/j.pmedr.2026.103434 (PMC12993333; doi:10.1016/j.pmedr.2026.103434)
Supplement: Supplementary file 1 — Supplementary material [file mmc1.docx]

**APPENDIX**

**Table A.1:** Differences in baseline outcomes and demographics between patients with complete and missing HbA1c data at 12-month follow-up, South Carolina, July 12, 2021- December 31, 2022 with 12-month follow-up

**Table A.2:** Differences in baseline outcomes and demographics between patients with complete and missing blood pressure data at 12-month follow-up, South Carolina, July 12, 2021- December 31, 2022 with 12-month follow-up

**Table A.3:** Differences in baseline outcomes and demographics between patients with complete and missing BMI data at 12-month follow-up, South Carolina, July 12, 2021- December 31, 2022 with 12-month follow-up

**Table A.4:** Differences in baseline outcomes and demographics between patients with complete and missing quality of life data at 12-month follow-up, South Carolina, July 12, 2021- December 31, 2022 with 12-month follow-up

**Table A.5:** Multiply imputed regression models depicting associations between clinical outcomes and quality of life and navigation intensity for resource navigator program participants and matched controls in South Carolina, July 12, 2021- December 31, 2022 with 12-month follow-up

**Table A.6:** Marginal effects of mean contact duration on predicted clinical outcomes, quality of life, and healthcare charges (from two-part models) over 12 months using multiply imputed data for resource navigator program participants and matched controls in South Carolina, July 12, 2021- December 31, 2022 with 12-month follow-up

**Table A.1: Differences in baseline outcomes and demographics between patients with complete and missing HbA1c data at 12-month follow-up, South Carolina, July 12, 2021- December 31, 2022 with 12-month follow-up**

| **Outcomes/**  **patient demographics** | | **Complete data** | | **Missing data** | | **Mean diff. (SE)** | **Test value**^†^**,**  **t (df)**  **or**  $\boldsymbol{x}^{\boldsymbol{2}}$ **(df)** | **p** |
| --- | --- | --- | --- | --- | --- | --- | --- | --- |
|  |  | **n** | **Mean (SD) or proportion*** | **n** | **Mean (SD) or proportion*** |  |  |  |
| **All patients** | | | | | | | | |
| HbA1c | | 50 | 7.67 (2.00) | 141 | 7.42 (1.99) | 0.25 (0.33) | 0.75 (189) | 0.46 |
|  |  |  |  |  |  |  |  |  |
| Age | | 50 | 57.92 (14.24) | 141 | 53.90 (13.68) | 4.02 (2.28) | 1.77 (189) | 0.08 |
|  |  |  |  |  |  |  |  |  |
| Gender | | 50 |  | 141 |  |  | 1.15 (1) | 0.29 |
|  | Male | 19 | 38.00% | 42 | 29.79% |  |  |  |
|  | Female | 31 | 62.00% | 99 | 70.21% |  |  |  |
|  |  |  |  |  |  |  |  |  |
| Race/ethnicity | | 50 |  | 141 |  |  | 0.17 (1) | 0.68 |
|  | Black or African American | 21 | 42.00% | 64 | 45.39% |  |  |  |
|  | Other race^†^ | 29 | 58.00% | 77 | 54.61% |  |  |  |
|  |  |  |  |  |  |  |  |  |
| Primary payer | | 50 |  | 140 |  |  | 0.73 (3) | 0.87 |
|  | Private/commercial^‡^ | 12 | 24.00% | 39 | 27.86% |  |  |  |
|  | Medicaid^§^ | 15 | 30.00% | 45 | 32.14% |  |  |  |
|  | Medicare^\|\|^ | 17 | 34.00% | 39 | 27.86% |  |  |  |
|  | Other payer^¶^ | 6 | 12.00% | 17 | 12.14% |  |  |  |
|  |  |  |  |  |  |  |  |  |
| Number of comorbidities | | 50 |  | 141 |  |  | 2.88 (2) | 0.24 |
|  | 1 | 5 | 10.00% | 12 | 8.51% |  |  |  |
|  | 2 | 19 | 38.00% | 37 | 26.24% |  |  |  |
|  | 3+ | 26 | 52.00% | 92 | 65.25% |  |  |  |
| **Resource navigator group** | | | | | | | | |
| HbA1c | | 24 | 7.50 (1.81) | 69 | 7.81 (2.39) | -0.31 (0.54) | -0.57 (91) | 0.57 |
|  |  |  |  |  |  |  |  |  |
| Age | | 24 | 55.88 (17.55) | 69 | 56.07 (11.74) | -0.20 (3.19) | -0.06 (91) | 0.95 |
|  |  |  |  |  |  |  |  |  |
| Gender | | 24 |  | 69 |  |  | 1.01 (1) | 0.32 |
|  | Male | 10 | 41.67% | 21 | 30.43% |  |  |  |
|  | Female | 14 | 58.33% | 48 | 69.57% |  |  |  |
|  |  |  |  |  |  |  |  |  |
| Race/ethnicity | | 24 |  | 69 |  |  | 0.26 (1) | 0.61 |
|  | Black or African American | 9 | 37.50% | 30 | 43.48% |  |  |  |
|  | Other race^†^ | 15 | 62.50% | 39 | 56.52% |  |  |  |
|  |  |  |  |  |  |  |  |  |
| Primary payer | | 24 |  | 68 |  |  | 0.87 (3) | 0.83 |
|  | Private/commercial^‡^ | 6 | 25.00% | 22 | 32.35% |  |  |  |
|  | Medicaid^§^ | 8 | 33.33% | 22 | 32.35% |  |  |  |
|  |  |  |  |  |  |  |  |  |
|  | Medicare^\|\|^ | 7 | 29.17% | 19 | 27.94% |  |  |  |
|  | Other payer^¶^ | 3 | 12.50% | 5 | 7.35% |  |  |  |
|  |  |  |  |  |  |  |  |  |
| Number of comorbidities | | 24 |  | 69 |  |  | 4.12 (2) | 0.13 |
|  | 1 | 3 | 12.50% | 4 | 5.80% |  |  |  |
|  | 2 | 9 | 37.50% | 15 | 21.74% |  |  |  |
|  | 3+ | 12 | 50.00% | 50 | 72.46% |  |  |  |
| **Control group** | | | | | | | | |
| HbA1c | | 26 | 7.82 (2.19) | 72 | 7.05 (1.43) | **0.77 (0.38)** | **2.02 (96)** | **0.05** |
|  |  |  |  |  |  |  |  |  |
| Age | | 26 | 59.81 (10.31) | 72 | 51.82 (15.10) | **7.99 (3.21)** | **2.49 (96)** | **0.01** |
|  |  |  |  |  |  |  |  |  |
| Gender | | 26 |  | 72 |  |  | 0.27 (1) | 0.61 |
|  | Male | 9 | 34.62% | 21 | 29.17% |  |  |  |
|  | Female | 17 | 65.38% | 51 | 70.83% |  |  |  |
|  |  |  |  |  |  |  |  |  |
| Race/ethnicity | | 26 |  | 72 |  |  | 0.01 (1) | 0.93 |
|  | Black or African American | 12 | 46.15% | 34 | 47.22% |  |  |  |
|  | Other race^†^ | 14 | 53.85% | 38 | 52.78% |  |  |  |
|  |  |  |  |  |  |  |  |  |
| Primary payer | | 26 |  | 72 |  |  | 1.20 (3) | 0.75 |
|  | Private/commercial^‡^ | 6 | 23.08% | 17 | 23.61% |  |  |  |
|  | Medicaid^§^ | 7 | 26.92% | 23 | 31.94% |  |  |  |
|  | Medicare^\|\|^ | 10 | 38.46% | 20 | 27.78% |  |  |  |
|  | Other payer^¶^ | 3 | 11.54% | 12 | 16.67% |  |  |  |
|  |  |  |  |  |  |  |  |  |
| Number of comorbidities | | 26 |  | 72 |  |  | 0.65 (2) | 0.72 |
|  | 1 | 2 | 7.69% | 8 | 11.11% |  |  |  |
|  | 2 | 10 | 38.46% | 22 | 30.56% |  |  |  |
|  | 3+ | 14 | 53.85% | 42 | 58.33% |  |  |  |

*Mean (SD) values presented for continuous variables and proportions for categorical variables

^††^Differences between missing and complete data examined using t-test (for continuous variables) or $\boldsymbol{x}^{\boldsymbol{2}}$ test (for categorical variables)

^†^Other race includes White, Hispanic, American Indian or Alaska Native, Native Hawaiian or Other Pacific Islander, Asian, and individuals with two or more races, patient refused/unknown race

^‡^Private/commercial includes BlueCross BlueShield and Commercial

^§^Medicaid includes Medicaid and Medicaid MCO, and Managed Care

^||^Medicare includes Medicare, and Medicare Advantage

^¶^Other payer includes other, self-pay, pending Medicaid, and Tricare

**Table A.2: Differences in baseline outcomes and demographics between patients with complete and missing blood pressure data at 12-month follow-up, South Carolina, July 12, 2021- December 31, 2022 with 12-month follow-up**

| **Outcomes/**  **patient demographics** | | **Complete data** | | **Missing data** | | **Mean diff. (SE)** | **Test value**^††^**,**  **t (df)**  **or**  $\boldsymbol{x}^{\boldsymbol{2}}$ **(df)** | **p** |
| --- | --- | --- | --- | --- | --- | --- | --- | --- |
|  |  | **n** | **Mean (SD) or proportion*** | **n** | **Mean (SD) or proportion*** |  |  |  |
| **All patients** | | | | | | | | |
| Systolic blood pressure | | 166 | 133.59 (16.95) | 232 | 132.48 (19.49) | 1.11 (1.88) | 0.59 (396) | 0.55 |
| Diastolic blood pressure | | 166 | 81.04 (10.92) | 232 | 81.33 (14.15) | -0.29 (1.31) | -0.22 (396) | 0.83 |
|  | |  |  |  |  |  |  |  |
| Age | | 166 | 56.02 (13.57) | 232 | 55.81 (13.72) | 0.20 (1.39) | 0.15 (396) | 0.88 |
|  |  |  |  |  |  |  |  |  |
| Gender | | 166 |  | 232 |  |  | 0.60 (1) | 0.44 |
|  | Male | 49 | 29.52% | 77 | 33.19% |  |  |  |
|  | Female | 117 | 70.48% | 155 | 66.81% |  |  |  |
|  |  |  |  |  |  |  |  |  |
| Race/ethnicity | | 166 |  | 232 |  |  | 1.84 (1) | 0.18 |
|  | Black or African American | 88 | 53.01% | 107 | 46.12% |  |  |  |
|  | Other race^†^ | 78 | 46.99% | 125 | 53.88% |  |  |  |
|  |  |  |  |  |  |  |  |  |
| Primary payer | | 166 |  | 232 |  |  | 2.10 (3) | 0.55 |
|  | Private/commercial^‡^ | 47 | 28.31% | 74 | 31.90% |  |  |  |
|  | Medicaid^§^ | 45 | 27.11% | 66 | 28.45% |  |  |  |
|  | Medicare^\|\|^ | 53 | 31.93% | 59 | 25.43% |  |  |  |
|  | Other payer^¶^ | 21 | 12.65% | 33 | 14.22% |  |  |  |
|  |  |  |  |  |  |  |  |  |
| Number of comorbidities | | 166 |  | 232 |  |  | 3.81 (2) | 0.15 |
|  | 1 | 26 | 15.66% | 47 | 20.26% |  |  |  |
|  | 2 | 53 | 31.93% | 86 | 37.07% |  |  |  |
|  | 3+ | 87 | 52.41% | 99 | 42.67% |  |  |  |
| **Resource navigator group** | | | | | | | | |
| Systolic blood pressure | | 83 | 134.25 (16.65) | 116 | 134.87 (20.97) | -0.62 (2.77) | -0.22 (197) | 0.82 |
| Diastolic blood pressure | | 83 | 81.86 (13.21) | 116 | 81.47 (15.12) | 0.39 (2.06) | 0.19 (197) | 0.85 |
|  |  |  |  |  |  |  |  |  |
| Age | | 83 | 55.63 (13.87) | 116 | 56.34 (13.13) | -0.72 (1.93) | -0.37 (197) | 0.71 |
|  |  |  |  |  |  |  |  |  |
| Gender | | 83 |  | 116 |  |  | 0.10 (1) | 0.75 |
|  | Male | 29 | 34.94% | 38 | 32.76% |  |  |  |
|  | Female | 54 | 65.06% | 78 | 67.24% |  |  |  |
|  |  |  |  |  |  |  |  |  |
| Race/ethnicity | | 83 |  | 116 |  |  | 0.03 (1) | 0.86 |
|  | Black or African American | 39 | 46.99% | 53 | 45.69% |  |  |  |
|  | Other race^†^ | 44 | 53.01% | 63 | 54.31% |  |  |  |
|  |  |  |  |  |  |  |  |  |
| Primary payer | | 83 |  | 116 |  |  | 2.20 (3) | 0.53 |
|  | Private/commercial^‡^ | 19 | 22.89% | 37 | 31.90% |  |  |  |
|  | Medicaid^§^ | 27 | 32.53% | 31 | 26.72% |  |  |  |
|  | Medicare^\|\|^ | 26 | 31.33% | 32 | 27.59% |  |  |  |
|  | Other payer^¶^ | 11 | 13.25% | 16 | 13.79% |  |  |  |
|  |  |  |  |  |  |  |  |  |
| Number of comorbidities | | 83 |  | 116 |  |  | 1.37 (2) | 0.50 |
|  | 1 | 18 | 21.69% | 18 | 15.52% |  |  |  |
|  | 2 | 28 | 33.73% | 45 | 38.79% |  |  |  |
|  | 3+ | 37 | 44.58% | 53 | 45.69% |  |  |  |
| **Control group** | | | | | | | | |
| Systolic blood pressure | | 83 | 132.93 (17.32) | 116 | 130.09 (17.65) | 2.84 (2.52) | 1.13 (197) | 0.26 |
| Diastolic blood pressure | | 83 | 80.23 (8.01) | 116 | 81.20 (13.16) | -0.97 (1.63) | -0.60 (197) | 0.55 |
|  |  |  |  |  |  |  |  |  |
| Age | | 83 | 56.41 (13.34) | 116 | 55.28 (14.31) | 1.13 (2.00) | 0.56 (197) | 0.58 |
|  |  |  |  |  |  |  |  |  |
| Gender | | 83 |  | 116 |  |  | 2.10 (1) | 0.15 |
|  | Male | 20 | 24.10% | 39 | 33.62% |  |  |  |
|  | Female | 63 | 75.90% | 77 | 66.38% |  |  |  |
|  |  |  |  |  |  |  |  |  |
| Race/ethnicity | | 83 |  | 116 |  |  | 3.02 (1) | 0.08 |
|  | Black or African American | 49 | 59.04% | 54 | 46.55% |  |  |  |
|  | Other race^†^ | 34 | 40.96% | 62 | 53.45% |  |  |  |
|  |  |  |  |  |  |  |  |  |
| Primary payer | | 83 |  | 116 |  |  | 3.13 (3) | 0.37 |
|  | Private/commercial^‡^ | 28 | 33.73% | 37 | 31.90% |  |  |  |
|  | Medicaid^§^ | 18 | 21.69% | 35 | 30.17% |  |  |  |
|  | Medicare^\|\|^ | 27 | 32.53% | 27 | 23.28% |  |  |  |
|  | Other payer^¶^ | 10 | 12.05% | 17 | 14.66% |  |  |  |
|  |  |  |  |  |  |  |  |  |
| Number of comorbidities | | 83 |  | 116 |  |  | **10.8 (2)** | **0.01** |
|  | 1 | 8 | 9.64% | 29 | 25.00% |  |  |  |
|  | 2 | 25 | 30.12% | 41 | 35.34% |  |  |  |
|  | 3+ | 50 | 60.24% | 46 | 39.66% |  |  |  |

*Mean (SD) values presented for continuous variables and proportions for categorical variables

^††^Differences between missing and complete data examined using t-test (for continuous variables) or $\boldsymbol{x}^{\boldsymbol{2}}$ test (for categorical variables)

^†^Other race includes White, Hispanic, American Indian or Alaska Native, Native Hawaiian or Other Pacific Islander, Asian, and individuals with two or more races, patient refused/unknown race

^‡^Private/commercial includes BlueCross BlueShield and Commercial

^§^Medicaid includes Medicaid and Medicaid MCO, and Managed Care

^||^Medicare includes Medicare, and Medicare Advantage

^¶^Other payer includes other, self-pay, pending Medicaid, and Tricare

**Table A.3: Differences in baseline outcomes and demographics between patients with complete and missing BMI data at 12-month follow-up, South Carolina, July 12, 2021- December 31, 2022 with 12-month follow-up**

| **Outcomes/**  **patient demographics** | | **Complete data** | | **Missing data** | | **Mean diff. (SE)** | **Test value**^††^**,**  **t (df)**  **or**  $\boldsymbol{x}^{\boldsymbol{2}}$ **(df)** | **p** |
| --- | --- | --- | --- | --- | --- | --- | --- | --- |
|  |  | **n** | **Mean (SD) or proportion*** | **n** | **Mean (SD) or proportion*** |  |  |  |
| **All patients** | | | | | | | | |
| BMI | | 181 | 35.99 (9.62) | 255 | 35.10 (10.42) | 0.89 (0.98) | 0.91 (434) | 0.37 |
|  | |  |  |  |  |  |  |  |
| Age | | 181 | 55.08 (13.71) | 255 | 54.62 (14.18) | 0.47 (1.36) | 0.34(434) | 0.73 |
|  |  |  |  |  |  |  |  |  |
| Gender | | 181 |  | 255 |  |  | 0.20 (1) | 0.66 |
|  | Male | 56 | 30.94% | 84 | 32.94% |  |  |  |
|  | Female | 125 | 69.06% | 171 | 67.06% |  |  |  |
|  |  |  |  |  |  |  |  |  |
| Race/ethnicity | | 181 |  | 255 |  |  | 0.55 (1) | 0.46 |
|  | Black or African American | 91 | 50.28% | 119 | 46.67% |  |  |  |
|  | Other race^†^ | 90 | 49.72% | 136 | 53.33% |  |  |  |
|  |  |  |  |  |  |  |  |  |
| Primary payer | | 181 |  | 254 |  |  | 2.12 (3) | 0.55 |
|  | Private/commercial^‡^ | 49 | 27.07% | 78 | 30.71% |  |  |  |
|  | Medicaid^§^ | 54 | 29.83% | 80 | 31.50% |  |  |  |
|  | Medicare^\|\|^ | 53 | 29.28% | 59 | 23.23% |  |  |  |
|  | Other payer^¶^ | 25 | 13.81% | 37 | 14.57% |  |  |  |
|  |  |  |  |  |  |  |  |  |
| Number of comorbidities | | 181 |  | 255 |  |  | 2.25 (2) | 0.32 |
|  | 1 | 35 | 19.34% | 58 | 22.75% |  |  |  |
|  | 2 | 57 | 31.49% | 90 | 35.29% |  |  |  |
|  | 3+ | 89 | 49.17% | 107 | 41.96% |  |  |  |
| **Resource navigator group** | | | | | | | | |
| BMI | | 90 | 36.51 (9.49) | 127 | 34.06 (9.72) | 2.45 (1.33) | 1.85 (215) | 0.07 |
|  |  |  |  |  |  |  |  |  |
| Age | | 90 | 54.50 (14.11) | 127 | 55.75 (13.26) | -1.25 (1.88) | -0.67 (215) | 0.51 |
|  |  |  |  |  |  |  |  |  |
| Gender | | 90 |  | 127 |  |  | 0.07 (1) | 0.80 |
|  | Male | 32 | 35.56% | 43 | 33.86% |  |  |  |
|  | Female | 58 | 64.44% | 84 | 66.14% |  |  |  |
|  |  |  |  |  |  |  |  |  |
| Race/ethnicity | | 90 |  | 127 |  |  | 0.03 (1) | 0.86 |
|  | Black or African American | 40 | 44.44% | 58 | 45.67% |  |  |  |
|  | Other race^†^ | 50 | 55.56% | 69 | 54.33% |  |  |  |
|  |  |  |  |  |  |  |  |  |
| Primary payer | | 90 |  | 126 |  |  | 2.03 (3) | 0.57 |
|  | Private/commercial^‡^ | 20 | 22.22% | 39 | 30.95% |  |  |  |
|  | Medicaid^§^ | 30 | 33.33% | 37 | 29.37% |  |  |  |
|  | Medicare^\|\|^ | 26 | 28.89% | 32 | 25.40% |  |  |  |
|  | Other payer^¶^ | 14 | 15.56% | 18 | 14.29% |  |  |  |
|  |  |  |  |  |  |  |  |  |
| Number of comorbidities | | 90 |  | 127 |  |  | 1.75 (2) | 0.42 |
|  | 1 | 23 | 255.6% | 23 | 18.11% |  |  |  |
|  | 2 | 30 | 333.3% | 47 | 37.01% |  |  |  |
|  | 3+ | 37 | 41.11% | 57 | 44.88% |  |  |  |
| **Control group** | | | | | | | | |
| BMI | | 91 | 35.48 (9.76) | 128 | 36.14 (11.0) | -0.66 (1.44) | -0.46 (217) | 0.65 |
|  |  |  |  |  |  |  |  |  |
| Age | | 91 | 55.66 (13.4) | 128 | 53.49 (15.0) | 2.17 (1.97) | 1.10 (217) | 0.27 |
|  |  |  |  |  |  |  |  |  |
| Gender | | 91 |  | 128 |  |  | 0.82 (1) | 0.37 |
|  | Male | 24 | 26.37% | 41 | 32.03% |  |  |  |
|  | Female | 67 | 73.63% | 87 | 67.97% |  |  |  |
|  |  |  |  |  |  |  |  |  |
| Race/ethnicity | | 91 |  | 128 |  |  | 1.50 (1) | 0.22 |
|  | Black or African American | 51 | 56.04% | 61 | 47.66% |  |  |  |
|  | Other race^†^ | 40 | 43.96% | 67 | 52.34% |  |  |  |
|  |  |  |  |  |  |  |  |  |
| Primary payer | | 91 |  | 128 |  |  | 2.82 (3) | 0.42 |
|  | Private/commercial^‡^ | 29 | 31.87% | 39 | 30.47% |  |  |  |
|  | Medicaid^§^ | 24 | 26.37% | 43 | 33.59% |  |  |  |
|  | Medicare^\|\|^ | 27 | 29.67% | 27 | 21.09% |  |  |  |
|  | Other payer^¶^ | 11 | 12.09% | 19 | 14.84% |  |  |  |
|  |  |  |  |  |  |  |  |  |
| Number of comorbidities | | 91 |  | 128 |  |  | **8.96 (2)** | **0.01** |
|  | 1 | 12 | 13.19% | 35 | 27.34% |  |  |  |
|  | 2 | 27 | 29.67% | 43 | 33.59% |  |  |  |
|  | 3+ | 52 | 57.14% | 50 | 39.06% |  |  |  |

*Mean (SD) values presented for continuous variables and proportions for categorical variables

^††^Differences between missing and complete data examined using t-test (for continuous variables) or $\boldsymbol{x}^{\boldsymbol{2}}$ test (for categorical variables)

^†^Other race includes White, Hispanic, American Indian or Alaska Native, Native Hawaiian or Other Pacific Islander, Asian, and individuals with two or more races, patient refused/unknown race

^‡^Private/commercial includes BlueCross BlueShield and Commercial

^§^Medicaid includes Medicaid and Medicaid MCO, and Managed Care

^||^Medicare includes Medicare, and Medicare Advantage

^¶^Other payer includes other, self-pay, pending Medicaid, and Tricare

**Table A.4: Differences in baseline outcomes and demographics between patients with complete and missing quality of life data at 12-month follow-up, South Carolina, July 12, 2021- December 31, 2022 with 12-month follow-up**

| **Outcomes/**  **patient demographics** | | **Complete data** | | **Missing data** | | **Mean diff. (SE)** | **Test value**^††^**,**  **t (df)**  **or**  $\boldsymbol{x}^{\boldsymbol{2}}$ **(df)** | **p** |
| --- | --- | --- | --- | --- | --- | --- | --- | --- |
|  |  | **n** | **Mean (SD) or proportion*** | **n** | **Mean (SD) or proportion*** |  |  |  |
| EQ-5D-5L score | | 67 | 0.64 (0.34) | 150 | 0.68 (0.30) | -0.04 (0.05) | -0.83 (215) | 0.41 |
|  | |  |  |  |  |  |  |  |
| Age | | 67 | 57.04 (13.77) | 150 | 54.31 (13.77) | 2.91 (2.02) | 1.44 (215) | 0.15 |
|  |  |  |  |  |  |  |  |  |
| Gender | | 67 |  | 150 |  |  | 2.24 (1) | 0.14 |
|  | Male | 28 | 41.79% | 47 | 31.33% |  |  |  |
|  | Female | 39 | 58.21% | 103 | 68.67% |  |  |  |
|  |  |  |  |  |  |  |  |  |
| Race/ethnicity | | 67 |  | 150 |  |  | 1.22 (1) | 0.27 |
|  | Black or African American | 34 | 50.75% | 64 | 42.67% |  |  |  |
|  | Other race^†^ | 33 | 49.25% | 86 | 57.33% |  |  |  |
|  |  |  |  |  |  |  |  |  |
| Primary payer | | 67 |  | 149 |  |  | 3.55 (3) | 0.31 |
|  | Private/commercial^‡^ | 15 | 22.39% | 45 | 30.20% |  |  |  |
|  | Medicaid^§^ | 18 | 26.87% | 48 | 32.21% |  |  |  |
|  | Medicare^\|\|^ | 21 | 31.34% | 37 | 24.83% |  |  |  |
|  | Other payer^¶^ | 13 | 19.40% | 19 | 12.75% |  |  |  |
|  |  |  |  |  |  |  |  |  |
| Number of comorbidities | | 67 |  | 150 |  |  | 0.13 (2) | 0.94 |
|  | 1 | 13 | 19.40% | 32 | 21.33% |  |  |  |
|  | 2 | 25 | 37.31% | 53 | 35.33% |  |  |  |
|  | 3+ | 29 | 43.28% | 65 | 43.33% |  |  |  |

*Mean (SD) values presented for continuous variables and proportions for categorical variables

^††^Differences between missing and complete data examined using t-test (for continuous variables) or $\boldsymbol{x}^{\boldsymbol{2}}$ test (for categorical variables)

^†^Other race includes White, Hispanic, American Indian or Alaska Native, Native Hawaiian or Other Pacific Islander, Asian, and individuals with two or more races, patient refused/unknown race

^‡^Private/commercial includes BlueCross BlueShield and Commercial

^§^Medicaid includes Medicaid and Medicaid MCO, and Managed Care

^||^Medicare includes Medicare, and Medicare Advantage

^¶^Other payer includes other, self-pay, pending Medicaid, and Tricare

**Table A.5: Multiply imputed regression models depicting associations between clinical outcomes and quality of life and navigation intensity for resource navigator program participants and matched controls in South Carolina, July 12, 2021- December 31, 2022 with 12-month follow-up**

|  |  | **(1)** | **(2)** | **(3)** | **(4)** | **(5)** |
| --- | --- | --- | --- | --- | --- | --- |
| **VARIABLES** | | **HbA1c**  **Mean**  **(Std. err.)** | **SBP**  **Mean**  **(Std. err.)** | **DBP Mean**  **(Std. err.)** | **BMI**  **Mean**  **(Std. err.)** | **QOL^#^**  **Mean**  **(Std. err.)** |
| Contact frequency (ref. = low) | |  |  |  |  |  |
|  | High | 0.16 | 3.00 | 1.03 | 1.00 | -0.04 |
|  |  | (0.42) | (2.47) | (1.69) | (1.30) | (0.03) |
|  | |  |  |  |  |  |
| Mean contact duration (ref. <6 minutes) | |  |  |  |  |  |
|  | 6-8 minutes | -0.17 | -4.41 | -3.94 | 0.66 | -0.15** |
|  |  | (0.56) | (3.40) | (2.36) | (1.56) | (0.05) |
|  | 8+ minutes | -0.55 | -0.41 | 1.50 | 3.08 | -0.16** |
|  |  | (0.59) | (3.33) | (2.27) | (1.72) | (0.05) |
|  |  |  |  |  |  |  |
| Time (ref. = baseline) | |  |  |  |  |  |
|  | 12 months | 0.17 | -1.24 | -1.55 | -0.31 | 0.01 |
|  |  | (0.31) | (1.71) | (0.91) | (0.33) | (0.05) |
|  |  |  |  |  |  |  |
| Mean contact duration × time (ref.= <6 minutes × baseline) | | |  |  |  |  |
|  | 6-8 minutes × 12 months | -0.07  (0.55) | -2.78 (2.94) | -0.31  (2.19) | -0.25  (0.56) | 0.07  (0.07) |
|  | 8+ minutes × 12 months | 0.04  (0.50) | -1.92  (3.72) | -1.08  (1.98) | 0.27  (0.49) | 0.06  (0.07) |
|  |  |  |  |  |  |  |
| Group (ref. = control) | |  |  |  |  |  |
|  | Intervention | 0.57  (0.49) | 3.68  (3.02) | 1.07  (2.07) | -2.10  (1.56) | - |
|  |  |  |  |  |  |  |
| *Patient demographics* | |  |  |  |  |  |
| Age | | -0.01 | -0.02 | -0.19** | -0.28** | -0.00 |
|  |  | (0.01) | (0.07) | (0.05) | (0.04) | (0.00) |
|  |  |  |  |  |  |  |
| Gender (ref.=female) | |  |  |  |  |  |
| Male | | 0.20  (0.34) | 3.28 (2.04) | 1.62  (1.15) | -3.22**  (0.99) | 0.00  (0.05) |
|  |  |  |  |  |  |  |
| Race/ethnicity (ref. = Black/African American) | | |  |  |  |  |
|  | Other race^†^ | -0.35 | -2.05 | -1.91 | -1.68 | -0.07 |
|  |  | (0.35) | (1.91) | (1.18) | (0.94) | (0.04) |
|  |  |  |  |  |  |  |
| Primary payer (ref. = Private/commercial^‡^) | | |  |  |  |  |
|  | Medicaid^§^ | -0.03 | 4.74 | 3.77** | -2.04 | -0.09 |
|  |  | (0.41) | (2.41) | (1.40) | (1.18) | (0.06) |
|  | Medicare^\|\|^ | -0.81 | 1.47 | -3.41* | 1.41 | -0.10 |
|  |  | (0.42) | (2.32) | (1.62) | (1.31) | (0.06) |
|  | Other payer^¶^ | -0.75 | 2.91 | 2.22 | -0.94 | -0.10 |
|  |  | (0.65) | (2.94) | (1.82) | (1.61) | (0.06) |
|  |  |  |  |  |  |  |
| No. of comorbidities (ref. = 1) | |  |  |  |  |  |
|  | 2 | -0.28 | 0.67 | -1.61 | 2.87* | -0.11* |
|  |  | (0.58) | (2.61) | (1.57) | (1.25) | (0.05) |
|  | 3+ | 0.05 | -0.33 | -2.31 | 1.84 | -0.14** |
|  |  | (0.5) | (2.58) | (1.52) | (1.25) | (0.05) |
|  |  |  |  |  |  |  |
| Constant | | 7.81** | 129.21** | 82.58** | 36.09** | 1.00** |
|  |  | (0.63) | (2.46) | (1.44) | (1.27) | (0.06) |
|  |  |  |  |  |  |  |
| No. of observations | | 434 | 796 | 796 | 874 | 432 |
| No. of groups | | 217 | 398 | 398 | 437 | 216 |
| F-test | | F(15,549.40)=0.96 | F(15,1213.90)=1.45 | F(15,1691.70)=7.15 | F(15,4242.80)=4.98 | F(14,586.70)=2.44 |
| Prob > F | | 0.50 | 0.12 | 0.00 | 0.00 | 0.00 |

Note: SBP = systolic blood pressure, DBP = diastolic blood pressure, BMI = body mass index, QOL = quality of life

Standard errors in parentheses

** p<0.01, * p<0.05

^†^Other race includes White, Hispanic, American Indian or Alaska Native, Native Hawaiian or Other Pacific Islander, Asian, and individuals with two or more races, patient refused/unknown race

^‡^Private/commercial includes BlueCross BlueShield and Commercial

^§^Medicaid includes Medicaid and Medicaid MCO, and Managed Care

^||^Medicare includes Medicare, and Medicare Advantage

^¶^Other payer includes other, self-pay, pending Medicaid, and Tricare
^#^Model includes only intervention group patients as QOL data was collected from exclusively from this group. A group indicator variable was therefore not applicable in this model specification.

**Table A.6: Marginal effects of mean contact duration on predicted clinical outcomes and quality of life over 12 months for multiply imputed data for resource navigator program participants and matched controls in South Carolina, July 12, 2021- December 31, 2022 with 12-month follow-up**

| **Outcomes** | | **Average-adjusted predicted means (SE)** | | |  | **Between group difference**  **Mean (SE)** | | **Within-group difference**  **Mean (SE)** |
| --- | --- | --- | --- | --- | --- | --- | --- | --- |
|  |  | **Baseline** | **12 months** | |  | **Baseline** | **12 months** |  |
| HbA1c (%) | |  |  | |  |  |  |  |
|  | <6 minutes | 7.57 (0.25) | 7.74 (0.33) | |  | ref. | ref. | 0.17 (0.31) |
|  | 6-8 minutes | 7.40 (0.43) | 7.50 (0.50) | |  | -0.14 (0.56) | -0.24 (0.69) | 0.10 (0.44) |
|  | 8+ minutes | 7.02 (0.43) | 7.23 (0.46) | |  | -0.55 (0.59) | -0.51 (0.59) | 0.21 (0.45) |
|  |  |  |  | |  |  |  |  |
| SBP (mmHg) | |  |  | |  |  |  |  |
|  | <6 minutes | 133.82 (1.44) | 132.57 (1.77) | |  | ref. | ref. | -1.24 (1.71) |
|  | 6-8 minutes | 129.41 (2.67) | 125.39 (2.85) | |  | -4.41 (3.40) | -7.19 (3.34)* | -4.02 (2.82) |
|  | 8+ minutes | 133.41 (2.53) | 130.25 (2.77) | |  | -0.41 (3.33) | -2.33 (3.80) | -3.17 (2.98) |
|  |  |  |  | |  |  |  |  |
| DBP (mmHg) | |  |  | |  |  |  |  |
|  | <6 minutes | 81.63 (0.93) | 80.08 (1.06) | |  | ref. | ref. | -1.55 (0.91) |
|  | 6-8 minutes | 77.69 (1.88) | 75.83 (2.09) | |  | -3.94 (2.36) | -4.25 (2.61) | -1.86 (2.03) |
|  | 8+ minutes | 83.13 (1.73) | 80.50 (2.06) | |  | 1.50 (2.27) | 0.42 (2.58) | -2.63 (1.80) |
|  |  |  |  | |  |  |  |  |
| BMI | |  |  | |  |  |  |  |
|  | <6 minutes | 34.81 (0.70) | 34.50 (0.72) | |  | ref. | ref. | -0.31 (0.33) |
|  | 6-8 minutes | 35.47 (1.19) | 34.90 (1.15) | |  | 0.66 (1.56) | 0.40 (1.55) | -0.56 (0.44) |
|  | 8+ minutes | 37.89 (1.34) | 37.85 (1.35) | |  | 3.08 (1.72) | 3.35 (1.70)* | -0.04 (0.46) |
|  |  |  |  | |  |  |  |  |
| QOL | |  | |  | | |  |  |
|  | <6 minutes | 0.78 (0.04) | 0.79 (0.045) | |  | ref. | ref. | 0.01 (0.05) |
|  | 6-8 minutes | 0.63 (0.04) | 0.71 (0.04) | |  | -0.15 (0.05)** | -0.08 (0.05) | 0.09 (0.05) |
|  | 8+ minutes | 0.62 (0.04) | 0.6 (0.04) | |  | -0.16 (0.05)** | -0.10 (0.06) | 0.08 (0.05) |

Note: BMI=Body mass index, DBP=diastolic blood pressure, SBP=systolic blood pressure, QOL=quality of life
** p<0.01, * p<0.05
